# Nitrogen form plays an important role in the growth of moso bamboo (*Phyllostachys edulis*) seedlings

**DOI:** 10.7717/peerj.9938

**Published:** 2020-09-16

**Authors:** Na Zou, Ling Huang, Huijing Chen, Xiaofeng Huang, Qingni Song, Qingpei Yang, Tianchi Wang

**Affiliations:** 1 College of Landscape and Art, Jiangxi Agricultural University, Nanchang, Jiangxi, China; 2 Jiangxi Provincial Key Laboratory for Bamboo Germplasm Resources and Utilization, Jiangxi Agricultural University, Nanchang, Jiangxi, China; 3 The New Zealand Institute for Plant & Food Research Limited (Plant & Food Research), Auckland, New Zealand

**Keywords:** *Phyllostachys edulis*, Nitrogen form, Ammonium, Nitrate, Potassium, Growth response, Nitrogen concentration

## Abstract

**Background:**

This study aimed to gain an understanding of the growth response of *Phyllostachys edulis* (moso bamboo) seedlings to nitrogen (N) and potassium (K) to benefit nutrient management practices and the design of proper fertilizer in nursery cultivation.

**Methods:**

An orthogonal array L_8_(4×2^4^) was used to study the effects of N forms (NH_4_^+^, NO_3_^−^), N concentrations (8, 32 mmol/L), and K^+^concentrations (0, 0.5, 1.5, 3 mmol/L) on seedling height, leaf number, chlorophyll content (SPAD value), biomass, root systems, and N content of *P. edulis* seedlings. Plants were grown in vermiculite under controlled greenhouse conditions.

**Results:**

Our study showed that N form played a significant role in the overall performance of *P. edulis* seedlings, followed by the effect of N and K^+^ concentrations. Among the N forms, NH_4_^+^ significantly improved the growth of *P. edulis* seedlings compared with NO_3_^−^. Seedling height, leaf number, chlorophyll SPAD value, biomass, and root system architecture (root length, root surface area, root volume, and root tips) were greater with 8 mmol/L of NH_4_^+^ treatments than with 32 mmol/L of NH_4_^+^treatments, whereas root diameter and N content of *P. edulis* seedlings were higher with 32 mmol/L of NH_4_^+^ than with 8 mmol/L of NH_4_^+^. K displayed inconsistent effects on the growth of *P. edulis* seedlings. Specifically, seedling height, leaf number, biomass and root volume increased when the K^+^ concentration was increased from 0 to 0.5 mmol/L, followed by a decrease when the K^+^ concentration was further increased from 0.5 to 3 mmol/L. Root average diameter of the seedlings was the highest with a K^+^ concentration of 1.5 mmol/L, and K had some inhibitory effects on the chlorophyll SPAD value of the seedlings. *P. edulis* seedlings performed well with 8 mmol/L NH_4_^+^and further tolerated a higher concentration of both NH_4_^+^ and NO_3_^−^, although excessive N could inhibit seedling growth. A lower concertation of K (≤ 0.5 mmol/L) promoted seedling growth and increasing K^+^ concentration in the nutrient solution did not alleviate the inhibitory effect of high N on the growth of *P. edulis* seedlings. Therefore, NH_4_^+^nitrogen as the main form of N fertilizer, together with a low concertation of K^+^, should be supplied in the cultivation and nutrient management practices of moso bamboo.

## Introduction

Moso bamboo (*Phyllostachys edulis*) is a species of large monopodial bamboo that is native to China and extensively cultivated throughout China, Japan, Korea, and Vietnam (Rao, Rao & Williams, 1998). Moso bamboo, which covers more than 3.87 hectares, representing 70% of the Chinese bamboo forest areas and 80% of the global distribution of moso bamboo, is the most important bamboo species in China (Song et al., 2016b). Moso bamboo can be planted by transplanting mother bamboo or planting seedling; the former method has generally been used for afforestation throughout China because vegetative propagation with mother bamboo typically takes only 5 years for it to form a grove compared with approximately 10 years by planting seedlings (Banik, 1980; Fu, 2000). However, both removal and replanting of the mother bamboo are labor intensive, high cost with low efficiency. In addition, human consumption of young edible shoots and environmental disturbance restrict the naturally rapid expansion of moso bamboo into a grove (Cai et al., 2008). In contrast, planting seedlings has many advantages, including easy handling, transporting, strong suitability and high survival rate with low cost (Fu, 2000). This provides a new approach for the regeneration and introduction of flowering bamboo forests. In fact, planting seedlings has been successfully applied in Guangxi Province, China to establish new bamboo groves (Qin, 2009; Chen, 2019). However, few studies have been conducted on the nutrient requirements of moso bamboo seedlings during cultivation and breeding. In addition, many bamboo forests are facing abandonment, with the forests potentially reverted to an unmanaged stand associated with a decline in soil organic matter accumulation and soil fertility (Christanty, Kimins & Mailly, 1997; Nakashima, 2001; Chen, Wang & Wang, 2016).

Nitrogen (N) is often an important factor for plant growth and productivity in terrestrial ecosystems. Under natural conditions, ammonium (NH_4_^+^) and nitrate (NO_3_^−^) are the two primary forms of inorganic N available to plants in soil (Xu, Fan & Miller, 2012). In well-aerated agricultural soils and disturbed or early-successional natural ecosystems, NO_3_^−^ is the major N source, whereas in flooded environments or acidic mature forests, NH_4_^+^ is the dominant N source (Glass et al., 2002; Britto & Kronzucker, 2006). Due to differences in environmental conditions, plant species, and the nutritional characteristics of N sources, plants have adapted to different N forms during long-term evolution and may show optimized growth with specific N forms (Britto & Kronzucker, 2013). For example, many conifers, ericaceous species and rice show improved growth with available NH_4_^+^, whereas some crops and early-successional pioneer species prefer NO_3_^−^ (Kronzucker, Siddiqi & Glass, 1997; Britto & Kronzucker, 2013). A previous study conducted by Li et al. (2014b) showed that the growth of *P. edulis* seedlings are slightly improved with available NO_3_^−^. However, Song et al. (2013) found that *P. edulis* tends to absorb NH_4_^+^under natural conditions. Gu et al. (2016) indicated that the N form preferred by *P. edulis* is related to the N concentration. Our study showed that under the low N concentrations (0.1, 0.4 mmol/L), there is no apparent N form preference, but the growth of bamboo seedlings especially the aboveground parts is improved with elevated NH_4_^+^ available from 2 to 40 mmol/L (Gu et al., 2016; Zou et al., 2020). Although NH_4_^+^can be used as a sole N source and an essential intermediate, it can also result in toxicity symptoms in many plant species, especially when high NH_4_^+^concentrations are provided as a sole N source or in combination with low levels of potassium (K) (Ten Hoopen et al., 2010). A previously study showed that shoot and root growth is significantly suppressed in cucumber grown with 10 mmol/L of NH_4_^+^ (Roosta & Schjoerring, 2008). Similar results have also been found in *Arabidopsis thaliana*, barley, tomato, and beans after high NH_4_^+^ treatments (Britto & Kronzucker, 2002; Britto & Kronzucker, 2006; Li et al., 2014a). In terms of moso bamboo, Li et al. (2014b) found that when the proportion of NH_4_^+^ exceeds 50% of the total N provided (40 mg/L), the growth of *P. edulis* seedlings is inhibited, and all of the seedlings eventually are died when NH_4_^+^ is supplied as a sole N source. However, Zou et al. (2020) showed that higher NH_4_^+^ concentrations (16∼40 mmol/L) are beneficial for the growth of aboveground organs, although root growth is suppressed to some extent with NH_4_^+^ levels ranging from 24 to 40 mmol/L. The contradictions in these studies indicate that N form preference and NH_4_^+^ tolerance of moso bamboo seedlings require further study.

K is another major plant nutrient that affects plant growth and metabolism (Wang et al., 2013). K plays a vital role in defending against biotic and abiotic stresses, and its role in the alleviation of NH_4_^+^ toxicity has been widely reported (Britto & Kronzucker, 2002; Szczerba et al., 2007; Wang et al., 2013). With similar hydration diameter charge and effects on membrane potential, K^+^ and NH_4_^+^ compete each other for the limited ion channel proteins on the cell membrane (Ten Hoopen et al., 2010; Kong et al., 2014; Coskun, Britto & Kronzucker, 2017). K^+^ can reduce the transport and accumulation of NH_4_^+^by direct competition during uptake and can also alleviate the rapid NH_4_^+^ cycling at the plasma membrane, thus reducing NH_4_^+^ toxicity. On the other hand, K^+^ can enhance NH_4_^+^ utilization by activating the enzymes of NH_4_^+^ assimilation and amino acid transport in plant cells (Wang, Siddiqi & Glass, 1996), which promotes NH_4_^+^ metabolism to reduce NH_4_^+^ toxicity (Ten Hoopen et al., 2010; Zhang et al., 2010).

Orthogonal arrays (often referred to as Taguchi methods) can be used to examine large numbers of factors in a much smaller number of experiments, allowing for the exploration of a unique subset of factor combinations. Therefore, it is a sophisticated time- and cost-efficient testing strategy (Lin, 1987; Qiao et al., 2013). Orthogonal experiments have been used to test the optimization of liquid fertilizer formulation, callus induction, plant regeneration medium in tissue culture and other hydroponic nutrient solution protocols for *Sorghum bicolor* (Gutiérrez-Miceli et al., 2008), *Dendrocalamus latiflorus* (Qiao et al., 2013), and *Ipomoea* spp*.* (Zhou & Lu, 2013). Using orthogonal arrays, we have studied the effects of different N and K^+^ concentrations and N form on the growth response and N acquisition of *P. edulis* seedlings. We have also investigated the extent to which the growth indices of *P. edulis* seedlings are related to each other. The overall aims of this study were to (1) further clarify the N form preference of *P. edulis* seedlings and (2) determine the effects of K on the response of moso bamboo to different N forms. This study presents the appropriate fertilization requirements of moso bamboo cultivation based on the experimental data.

## Materials and Methods

### Plant materials and growth conditions

Seeds of *P. edulis* were collected from GuanYang City, Guangxi Province, China and stored at 4 °C before sowing. The moso bamboo seeds were soaked overnight in water at 40 °C, shelled, sterilized by soaking in 20% NaClO for 20 min, rinsed in sterile water at least five times, and then germinated in plastic pots (diameter of 150 mm, height of 130 mm) filled with vermiculite. One month later, approximately five cm tall, three foliate seedlings were selected for different treatments.

### N and K treatments

L_8_ (4 × 2^4^) orthogonal arrays were employed to study K^+^ concentrations (0, 0.5, 1.5, 3 mmol/L), N forms (NH_4_^+^, NO_3_^−^), and N concentrations (8, 32 mmol/L) on seedling growth and N uptake (Table 1). The nutrient solution modified from Norisada & Kojima (2005) contains 2.5 mmol/L Ca_2_^+^ as CaCl_2_ ⋅ 2H_2_O, 0.25 mmol/L MgSO_4_ ⋅ 7H_2_O, 0.6 mmol/L Na_2_HPO_4_ ⋅ 10H_2_O, 0.01 mmol/L Fe-EDTA, 0.02 mmol/L H_3_BO_3_, 2 µmol/L MnCl_2_ ⋅ 4H_2_O, 2 µmol/L ZnSO_4_ ⋅ 7H_2_O, 2 µmol/L CuSO_4_ ⋅ 5H_2_O, 0.5 µmol/L Na_2_MoO_4_ ⋅ 2H_2_O, and 0.5 µmol/L CoCl_2_ ⋅ 6H_2_O. The K^+^, NH_4_^+^, and NO_3_^−^ were supplied by using KCl, (NH_4_)_2_SO_4_, and NaNO_3_, respectively. A nitrification inhibitor C_2_H_4_N_4_ (7 µM) was added to all treatments to prevent nitrification. One replicate consisted of six pots with two seedlings planted per pot, there were three replicates per treatment. Plants were grown in a greenhouse at 25/18 °C ± 3 °C day/night temperature, 65∼70% relative humidity, and 14/10 h day/night photoperiod. Pots were rotated every week to eliminate location effects. After treatment for 2 months, aboveground growth traits, root systems, and N content were analyzed.

**Table 1:** L_8_(4×2^4^) orthogonal array design of nutrient solution composition with the concentrations and forms of N and K.

Orthogonal combination	K concentration (mmol/L)	N form	N concentration (mmol/L)
1	0	NH_4_^+^	8
2	0	NO_3_^−^	32
3	0.5	NH_4_^+^	32
4	0.5	NO_3_^−^	8
5	1.5	NH_4_^+^	32
6	1.5	NO_3_^−^	8
7	3	NH_4_^+^	8
8	3	NO_3_^−^	32

### Growth analysis and root morphology

Height was measured with a ruler. The number of unfurled leaves above the cotyledonary node were counted. The chlorophyll content (SPAD value) of the leaves was determined with a chlorophyll meter (SPAD-502, Minolta). Root morphology, including root total length (RL), average diameter (AD), root surface area (RS), root volume (RV) and root tips were determined using an automatic scanning apparatus (EPSON color image scanner LA1600+, Toronto, Canada) equipped with WinRHIZO 2012 software (Regent Instruments, Quebec, Canada).

### Measurement of dry weight and N content

When the above experimental treatment was completed, plants were dried at 105 °C for 30 min, and then dried to a constant weight at 70 °C for biomass determination. The samples were digested with H_2_SO_4_-H_2_O_2_, and the total N content was determined according to the Kjeldahl method (Chen et al., 2013).

### Statistical analysis

The data were analyzed to determine the range (R and R’) of orthogonal tests using DPS7.05 statistical software (http://www.statforum.com). Using the same software, an analysis of variance (ANOVA) was conducted and post hoc comparisons were conducted using Duncan’s multiple comparison test with differences considered significant at *p* < 0.05.

## Results

### Effects of N and K on the growth of moso bamboo seedlings

**Figure 1:**
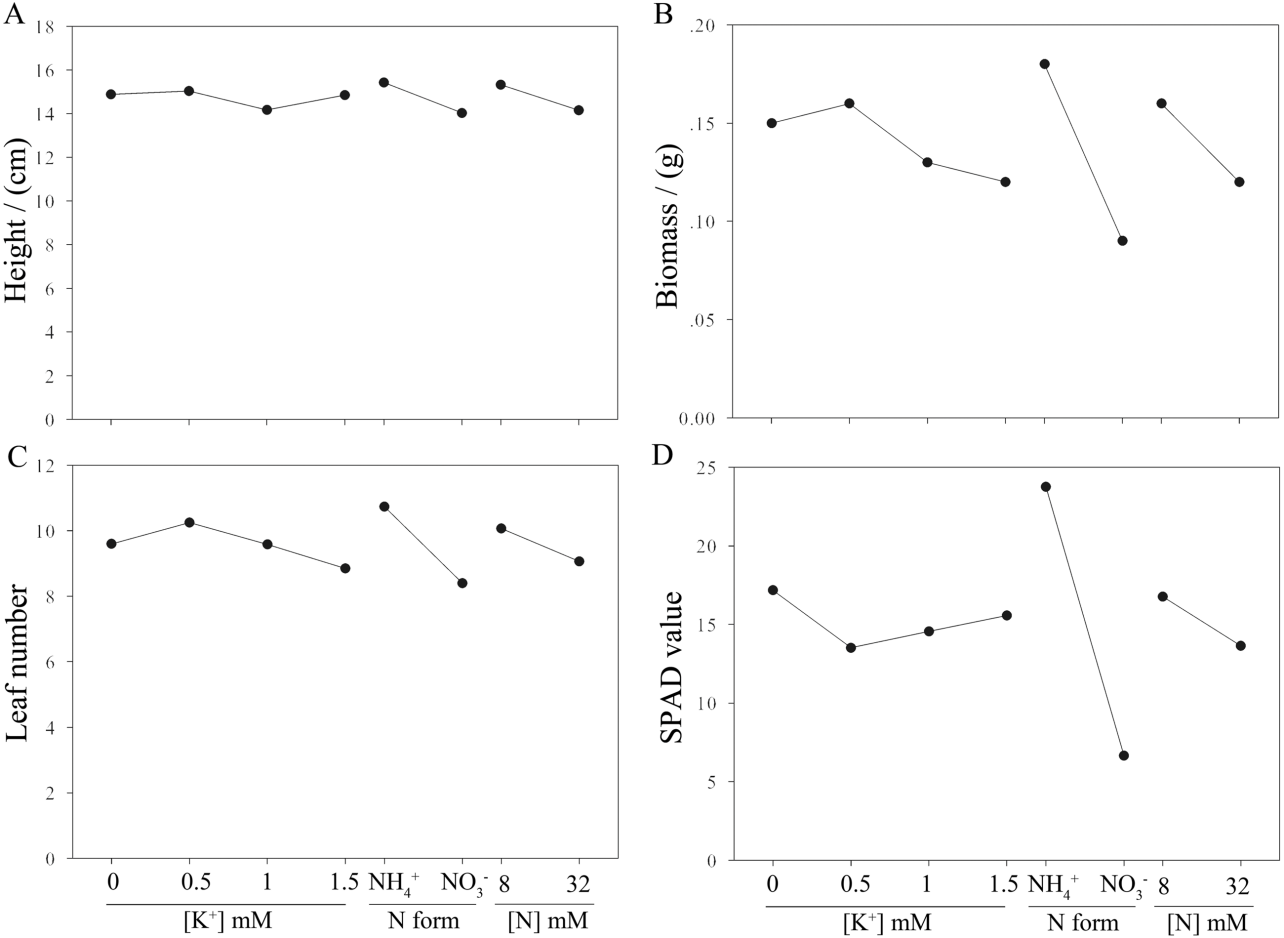
Effects of N and K on aboveground growth of moso bamboo seedlings. A to D indicates the variation tendency of seedling height (A), biomass (B), leaf number (C) and SPAD value (D) respectively to different K concentrations, N form and N concentrations.

The effects of N and K on bamboo seedlings are listed in Table 2. The range analysis (R’ values) of the orthogonal array indicated that N form had a significant impact on the seedling height, biomass, leaf number, and SPAD value of *P. edulis*. Impacts to a lesser extent were observed for N concentration, while K^+^ concentration had the least effect (R’_N form_ >  R’_N concentration_ >R’_K_^+^_concentration_, Table 3), suggesting that N form is more important than nitrogen and potassium concentration on growth of moso bamboo seedling. After two months of treatment with the different N forms (Table 1), the seedlings of moso bamboo performed better in the treatments of NH_4_^+^ (treatments 1, 3, 5, 7) than the treatments of NO_3_^−^ (treatments 2, 4, 6, 8) , and seedlings showed greener, heathier leaves and less necrosis in the NH_4_^+^ treatments than in the NO_3_^−^ treatments (Fig. S1). The growth index of seedling height, biomass, leaf number and SPAD value increased 9.98%, 100%, 27.86%, and 257.14%, respectively, by comparing average value of these parameters between the treatments of 1, 3, 5, 7 and the treatments of 2, 4, 6, 8 (Table 2, Fig. 1). For the N concentrations (Table 1), the results showed that seedling height, biomass, leaf number and SPAD value increased by 8.27%, 33.33%, 11.03%, and 22.96%, respectively (Fig. 1), with the nutrition solutions of 8 mmol/L of nitrogen (treatments 1, 4, 6, 7) compared to 32 mmol/L of nitrogen (treatments 2, 3, 5, 8; Table 2).

**Table 2:** N and K on growth, N content, and root architecture of moso bamboo seedlings. Analytical results are means ± SE (*n* = 36). Mean values followed by the same letter (a, b, c, d, e, or f) are not significantly different within the same column according to a Duncan’s multiple comparison test at *P* < 0.05.

Treatment	Mortality rate/(%)	Height (cm)	Leaf number	SPAD	Biomass (g)	N content (%)	Root length (cm)	Root surface area (cm^2^)	Root volume (cm^3^)	Average diameter (cm)	Root tips
1	8.00a	16.63a	11.44a	27.55a	0.22a	0.92ab	292.86a	30.80a	0.26a	0.34ab	1438.72a
2	0.00a	13.13b	7.75f	6.81d	0.08de	0.84bc	117.98cd	12.34cd	0.10c	0.33ab	612.75cd
3	0.00a	15.70ab	11.0ab	20.52c	0.20a	0.72bc	209.02b	23.81b	0.22ab	0.37ab	915.33b
4	0.00a	14.37ab	9.50de	6.49d	0.12cd	0.39d	186.95b	18.58bc	0.15bc	0.32b	1019.58b
5	0.00a	13.73b	10.17cd	21.51bc	0.14bc	1.14a	99.45d	11.59cd	0.11c	0.37a	393.25d
6	0.00a	14.60ab	9.00e	7.59d	0.11cde	0.60cd	176.63bc	18.13bc	0.15bc	0.32ab	812.17bc
7	0.00a	15.67ab	10.33bc	25.42ab	0.18ab	0.45d	196.28b	21.25b	0.18b	0.34ab	802.50bc
8	8.00a	14.03ab	7.36f	5.70d	0.07e	0.39d	77.79d	8.43d	0.07c	0.34ab	366.22d

**Table 3:** Range analysis of the concentrations and forms of N and K on the growth indices of the seedlings. R is the range of each factor level (R=*X*_max_−*X*_min_); R’ is the adjusted range.

Range and adjusted range of growth indices	K concentration	N form	N concentration
R_Seedling height_	0.87	1.40	1.17
R’_Seedling height_	0.55	1.99	1.66
R_Biomass_	0.04	0.09	0.03
R’_Biomass_	0.02	0.13	0.05
R_leaf number_	1.40	2.33	1
R’_leaf number_	0.89	3.31	1.42
R_SPAD__value_	3.68	17.10	3.13
R’_SPAD__value_	2.34	24.28	4.44
R_Root length_	68.39	59.57	87.12
R’_Root length_	43.52	84.58	123.71
R_Root surface area_	6.73	7.50	8.15
R’_Root surface area_	4.28	10.65	11.57
R_Root volume_	0.06	0.07	0.06
R’_Root volume_	0.04	0.10	0.08
R_Average diameter_	0.01	0.03	0.02
R’_Average diameter_	0.004	0.043	0.028
R_Root tips_	441.38	184.77	446.35
R’_Root tips_	280.89	262.37	633.82
R_N content_	0.75	0.46	0.06
R’_N content_	0.26	0.29	0.09

The analysis of variance (Table 4) showed that the N form and N concentration had significant effects on seedling height, biomass, leaf number and SPAD value. The K^+^ concentration also had significant effects on the leaf number of *P. edulis* seedlings. Therefore, moso bamboo seedlings treated with a normal nutrient solution supplemented with 8 mmol/L NH_4_^+^ and 0.5 mmol/L K^+^ would show overall better growth indices.

**Table 4:** Variance analysis of the concentrations and forms of N and K on the growth indices of the seedlings.

Source of variation	The test statistic F		
	K concentration	N form	N concentration
Height	0.53	6.94^*^	4.82^*^
Leaf number	8.65^**^	143.16^**^	26.29^**^
SPAD value	2.58	307.62^**^	10.29^*^
Biomass	2.70	61.25^**^	8.26^*^
Root length/(cm)	7.14^**^	18.34^**^	39.24^**^
Root surface area/(cm^2^)	5.71^**^	22.50^**^	26.59^**^
Root volume/(cm^3^)	3.94^*^	22.39^**^	14.22^**^
Average diameter/(cm)	0.16	7.72^*^	3.42
Root tips	16.26^**^	10.13^**^	59.13^**^
Nitrogen content	18.13^**^	21.62^**^	11.48^**^

**Notes.**

* indicates the significance level at *p* < 0.05.

** indicates the significance level at *P* < 0.01.

### Effects of different N and K on root system architecture of moso bamboo seedlings

**Figure 2:**
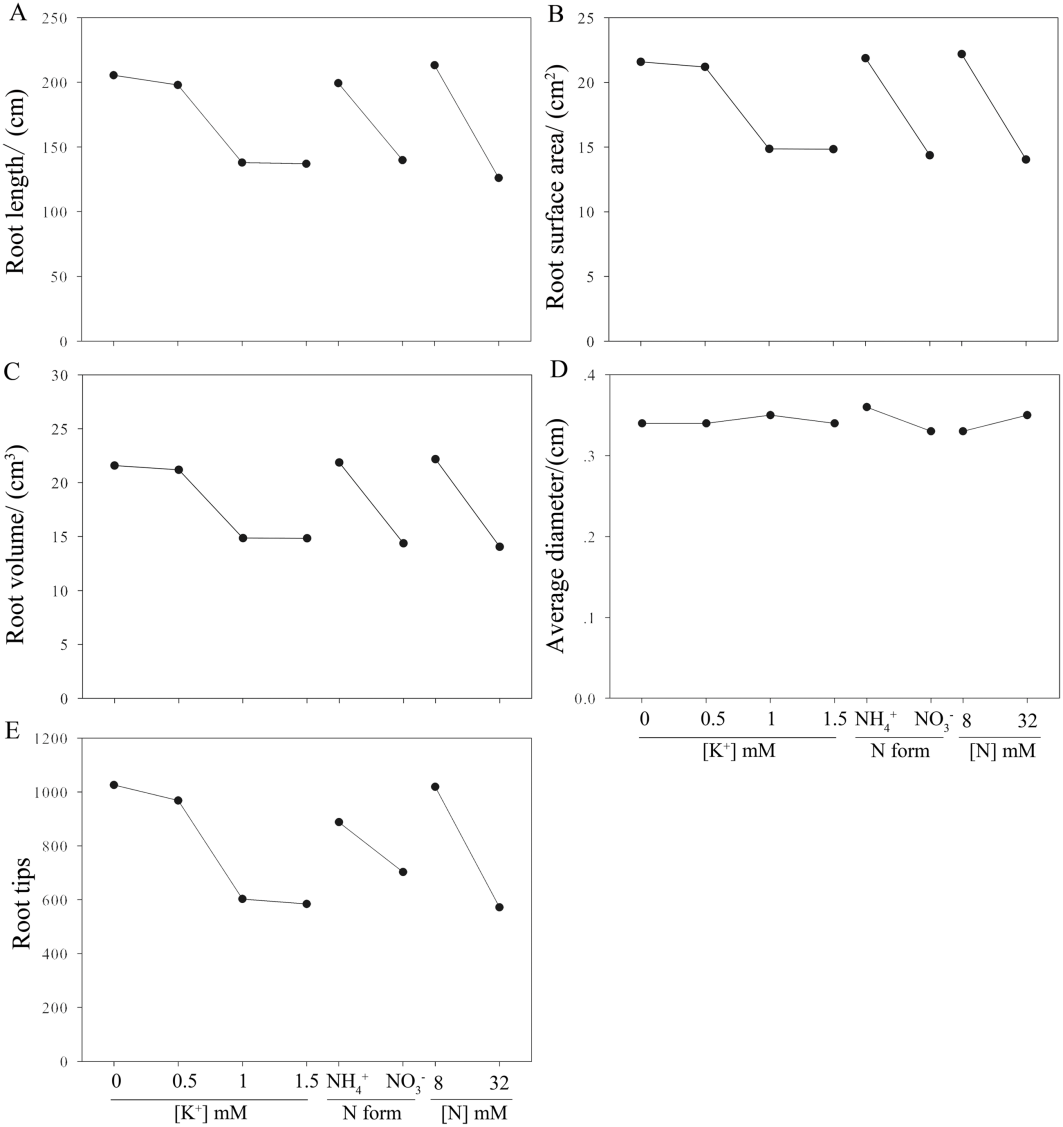
Effects of N and K on root system architecture of moso bamboo seedlings. A to E indicates root system architecture of root total length (A), root surface area (B), root volume (C), average diameter (D) and root tips (E) to different K concentrations, N form and N concentrations.

According to the resultant R’ values of root architecture parameters (Table 3), N form and N concentration (Table 1) played a significant role on the root growth of *P. edulis*, while K^+^ concentration showed the least effect. Root architecture parameters were improved with NH_4_^+^ compared to NO_3_^−^, and root traits were improved under the 8 mmol/L nitrogen (treatments 1, 4, 6 and 7; Tables 1 and 2) compared with the 32 mmol/L nitrogen (treatments 2, 3, 5 and 8; Tables 1 and 2), although the higher N concentration resulted in a greater increase in the root diameter of *P. edulis* (Table 2, Fig. 2). K had inconsistent effects on the root architecture of *P. edulis*. The total root length, root surface area and the root tips smaller with the increasing K^+^ concentrations. In contrast, the root volume and root diameter showed improved growth at a K^+^ concentration of 0.5 and 1.5 mmol/L, respectively (Table 2; Fig. 2).

The analysis of variance (Table 4) showed that K^+^ concentration, N form and N concentration had significant effects on most of the root system parameters. However, there was no evident influence of K^+^ and N concentrations on average root diameter. In general, *P. edulis* seedlings treated with 8 mmol/L NH_4_^+^ (treatment 1) showed significantly better root architecture parameters than seedlings cultured in the other combinations/treatments (Table 2).

### Effects of different N and K^+^ concentrations on the N content of moso bamboo seedlings

**Figure 3:**
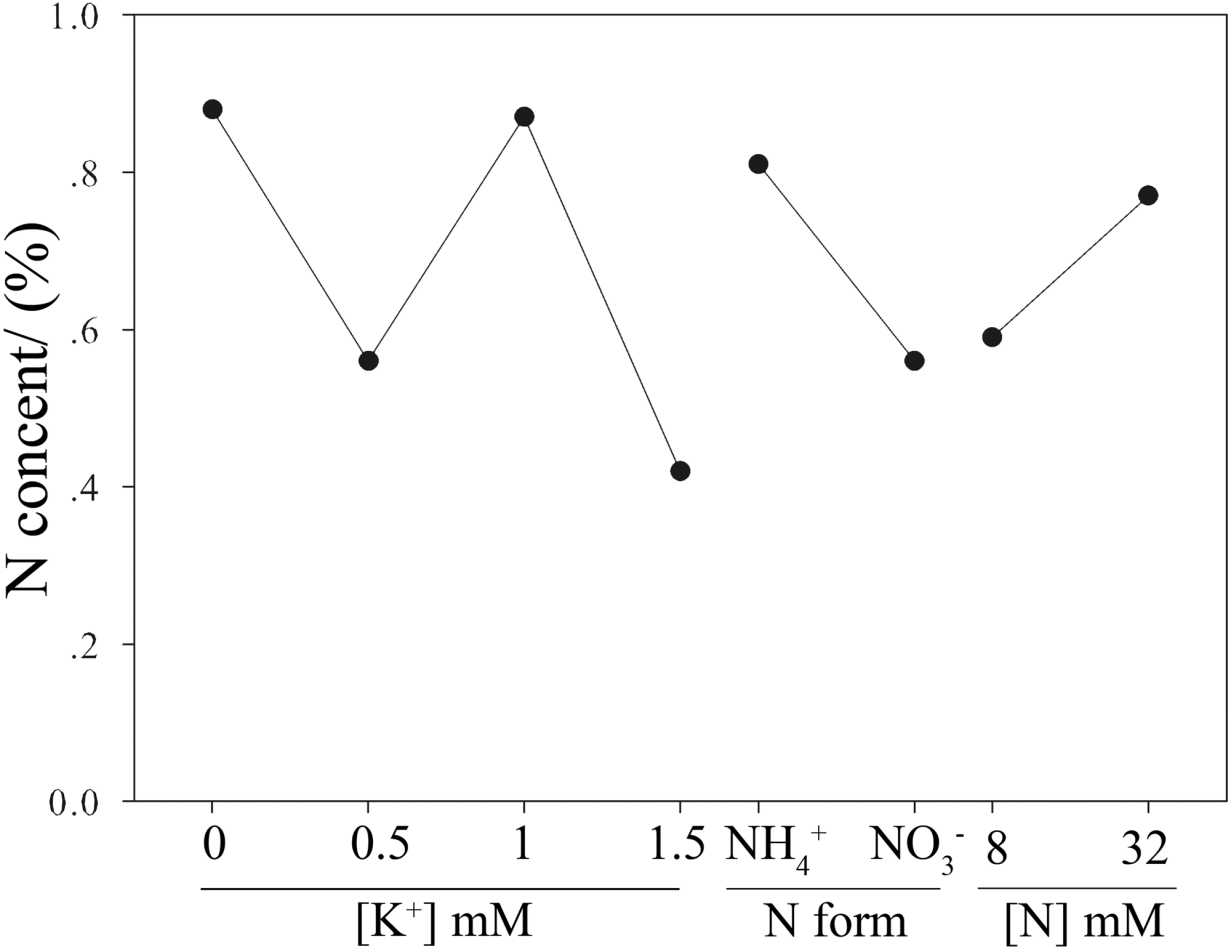
Effects of N and K on N content of moso bamboo seedlings.

According to the resultant R’ values, the N form and K^+^ concentration (Table 1) played a more significant role than N concentration on the N content of moso bamboo seedlings (R’_N form_ >  R’_K_^+^_concentration_ >  R’_N concentration_, Table 3). NH_4_^+^ was a better N form than the NO_3_^−^ for the N content of the seedlings, increasing 44.64% by comparing the average value of treatments 1, 3, 5, and 7 and treatments 2, 4, 6, and 8 (Tables 1 and 2, Fig. 3). The N content decreased when the K^+^ concentration increased from 0 to 0.5 mmol/L, whereas the N content increased when the K^+^ concentration further increased from 0.5 to 1.5 mmol/L. The optimal K^+^ concentration for the N content of moso bamboo seedlings was 0.5 to 1.5 mmol/L. The N content of the seedlings increased by 32.2% when the N concentration (Table 1) increased from 8 mmol/L (treatments 1, 4, 6 and 7) to 32 mmol/L (treatments 2, 3, 5 and 8; Tables 1 and 2, Fig. 3).

The analysis of variance showed that the effects of N form, N concentration, and K^+^ concentration (Table 1) on the N content of the seedlings were very significant (Table 4). Furthermore, the results indicate that moso bamboo seedlings had higher N content in treatment 5 (32 mmol/L NH_4_^+^ + 1.5 mmol/L K^+^) compared with the other treatments, except for treatment 1 (32 mmol/L NH_4_^+^ + 0 mmol/L K^+^) (Table 2).

### Correlation analysis of the growth indices of moso bamboo seedlings

The growth indices of seedling height, leaf number, SPAD value and biomass were positively correlated with each other, and the correlations were also positive for root system architecture parameters such as root length, root surface area, root volume and root tips (Table 5). Growth and biomass accumulation were positively correlated with the main root system architecture parameters, but negatively or not correlated with N content. There were no correlations between root system architecture and the N content of the seedlings (Table 5).

**Table 5:** Correlation analysis of the growth indices of the seedlings.

Correlations *n* = 8	Height	Leaf number	SPAD	Biomass	N content	Root length	Root surface	Root volume	Average diameter	Root tips
Height	1	–	–	–	–	–	–	–	–	–
Leaf number	.804^*^	1	–	–	–	–	–	–	–	–
SPAD	.727^*^	.926^**^	1	–	–	–	–	–	–	–
Biomass	.890^**^	.975^**^	.903^**^	1	–	–	–	–	–	–
N content	−0.053	0.395	0.459	0.296	1	–	–	–	–	–
Root length	.889^**^	.787^*^	0.582	.827^*^	0.042	1	–	–	–	–
Root surface	.917^**^	.832^*^	0.653	.884^**^	0.093	.992^**^	1	–	–	–
Root volume	.923^**^	.880^**^	.709^*^	.928^**^	0.157	.966^**^	.990^**^	1	–	–
Average diameter	0.155	0.5	0.588	0.448	0.554	−0.094	0.023	0.155	1	–
Root tips	.785^*^	0.696	0.418	0.701	0.002	.970^**^	.939^**^	.893^**^	−0.239	1

**Notes.**

* Correlation is significant at the 0.05 level (2-tailed).

** Correlation is significant at the 0.01 level (2-tailed).

## Discussion

### Moso bamboo seedlings showed improved growth under the NH_4_^+^ nitrogen form

All of the tested growth parameters and total N content were significantly increased with the NH_4_^+^ treatments compared to the NO_3_^−^ treatments, suggesting that the seedlings of moso bamboo prefer NH_4_^+^ (Figs. 1–3). Our results are consistent with those presented by Gu et al. (2016) and Zou et al. (2020) who showed that the growth of bamboo seedlings displayed a strong NH_4_^+^ preference. Similarly, Song et al. (2013) showed that N uptake in the field is mainly in the form of NH_4_^+^, accounting for 93.6% of the total inorganic N in bamboo-dominated forests. In addition, Ueda (1960) reported that the application of ammonium sulfate ((NH_4_)_2_SO_4_) strongly promotes the production of new culms in the first year after fertilization of *P. edulis* and *P. reticulata* groves. However, such results contradict the findings presented by Li et al. (2014b) that moso bamboo shows limited preference for NO_3_^−^. Considering the cultivation medium, pH and nutrient supply are different among these experiments (Li et al., 2014b; Gu et al., 2016; Zou et al., 2020), it is hypothesized that the interactions between plant acquisition of N and multiple environmental variables may produce a complex of effects that can greatly influence and shift plant growth responses to variable N sources (Britto & Kronzucker, 2013). Although environmental factors, such as variations in pH, can also affect preferences for N form (Britto & Kronzucker, 2013), it has been reported that the effect of N form on plant performance is independent of growth medium pH (3.8, 5.8) for the *P. edulis* (Gu et al., 2016). As pH differences among treatments were negligible in our study, we believe, therefore, that performance differences are attributable to the N form rather than to the pH. However, the reasons underlying the preference of NH_4_^+^ by *P. edulis* are poorly understood and may arise from the atrophied nitrate uptake systems in the roots of *P. edulis*, as there only six nitrate transporters compared to twenty ammonium transporters were identified in the *P. edulis* genome (Hou, 2020), which was similar to the “ammonium specialists” of many conifers (Kronzucker, Siddiqi & Glass, 1997). Furthermore, moso bamboo is mainly distributed in subtropical acidic soils of southern areas of China, with N mineralization dominated by ammonification and NH_4_^+^ is the dominant inorganic N form in the soil (Song et al., 2013; Song et al., 2016a; Li et al., 2017). Therefore, moso bamboo preferentially utilizing NH_4_^+^ may be due to an adaptation to the native N nutritional habitat presenting over the course of evolution, similar to most late-successional conifer species growing on acidic soils (Kronzucker, Siddiqi & Glass, 1997; Britto & Kronzucker, 2013).

In aboveground organs, N is a structural element of chlorophyll, which affects the formation of chloroplasts and the accumulation of chlorophyll (Tucker, 2004). A previous study has reported that the chlorophyll content is closely linked to the leaf N content, and N deficiency leads to loss of the green color in the leaves, a reduced leaf area and photosynthetic intensity (Bojović & Marković, 2009). In the present work, the green color in the leaves and leaf area of the seedlings treated with NO_3_^−^are decreased compared to those treated with NH_4_^+^ (Fig. S1), which may be associated with the leaf N content. However, there was no correlation between the total N content and chlorophyll content (SPAD value) or other growth characters (Table 5). One reason for this may be that we measured the N content of the whole plant instead of determining the N content of the root, stem, and leaf separately, so the total N content is inconsistent with the leaf N content. Alternatively, the correlation between the total N content and other growth characters may have not been established during our short-term experiment. Different forms of N have a large effect on leaf growth because N increases the leaf area of plants, chlorophyll content of leaf blades and photosynthetic rate, influencing photosynthesis (Li, Wang & Stewart, 2013). Therefore, the greater growth of moso bamboo seedlings with NH_4_^+^than with NO_3_^−^ might be also associated with the increased photosynthesis of the aboveground organs.

Size and architecture of the root system are important factors of nutrient acquisition efficiency as they ensure the total volume of soil explored by the plant, and the total surface of exchange between roots and the soil solution (Nacry, Bouguyon & Gojon, 2013). Root system architecture is highly plastic, strongly modulated by N availability. A change in the root system can greatly impact nutrient acquisition from soil. In the present study, N form has a dramatic impact on the root system architecture of moso bamboo seedlings. Accordingly, the overall plant height, leaf number and biomass could be improved with the greater root length, root surface area and root volume in the bamboo seedlings treated with NH_4_^+^ (Figs. 1 and 2), due to positive correlations between most parameters of root morphology and aboveground indices (Table 5).

### Moso bamboo suffers from the toxicity due to excessive NH_4_^+^

Although NH_4_^+^ is a preferred N source, excessive NH_4_^+^ such as 32 mmol/L (treatments 3 and 5), suppress aboveground growth, biomass accumulation and root system, while the total N content is increased by 35.77% compared to treatments with 8 mmol/L NH_4_^+^(treatments 1 and 7) (Table 2), suggesting that the *P. edulis* seedlings suffer from the toxicity due to excessive NH_4_^+^.

The uptake of NH_4_^+^ and NO_3_^−^ by the root system is determined by different affinities. In the low concentration range, the uptake is mediated by high-affinity transport systems (HATS), while under high concentrations (typically >0.5∼1.0 mmol/L), the activity of low affinity transport systems (LATS) takes over from HATS (Nacry, Bouguyon & Gojon, 2013). Unlike HATS, the NH_4_^+^ uptake mediated by the LATS is not saturated and poorly regulated. It generally shows a linear increase with the increase of the external concentration, when NH_4_^+^ absorbed by roots far exceeds the amount of assimilation, it will cause excessive levels of NH_4_^+^ and thus plant NH_4_^+^ toxicity (Givan, 1979; Glass et al., 2002; Nacry, Bouguyon & Gojon, 2013). On the other hand, with the high similarity in the charge, size and hydration energy between NH_4_^+^ and K^+^, the K^+^ion transporters and channels do not discriminate between NH_4_^+^ and K^+^. The NH_4_^+^ can be transported through K^+^ transporters and channels as well as nonselective cation channels (NSCC), which may also contribute to NH_4_^+^ toxicity, especially at low K^+^ levels (Ten Hoopen et al., 2010). Although moso bamboo showed growth suppression with the high NH_4_^+^ treatments, there was no difference in the survive rate compared to the 8 mmol/L NH_4_^+^ treatments (Table 2), indicating moso bamboo seedlings were tolerant to high concentrations of ammonium.

Balkos, Britto & Kronzucker (2010) and Li et al. (2012) have underscored the central importance of NH_4_^+^/K^+^ ratios to determine NH_4_^+^ toxicity and tolerance in plants. However, the alleviation of NH_4_^+^ toxicity by K^+^ addition is not obvious for *P. edulis* seedlings. Under high NH_4_^+^ conditions, elevated exogenous K^+^ from 0.5 mmol/L to 1.5 mmol/L (treatments 3 and 5) caused a further inhibition of plant growth and increased NH_4_^+^ accumulation in moso bamboo seedlings. The external K^+^ may not be high enough to alleviate NH_4_^+^ toxicity, as described previously for *Arabidopsis* treated with 5∼20 mmol/L KNO_3_ (Zou et al., 2012). Alternately, moso bamboo may have evolved mechanisms of vacuolar nitrogen storage and downward transport of nitrogen from aerial parts to roots, allowing the plant to maintain nitrogen homeostasis, by which they may survive exposure to potentially toxic NH_4_^+^ concentrations, similar to the findings from other studies (Britto et al., 2001; Kronzucker et al., 2001; Britto & Kronzucker, 2002). Further studies need to be conducted to determine whether there are other mechanisms and the exact mode of actions of K^+^ and NH_4_^+^ relevant to the toxicity and tolerance.

### Growth traits of moso bamboo are not improved by elevated K^+^

A previous study indicated that K^+^ is the major osmotically active cation contributing to the maintenance of root cell turgor and expansion, therefore plant roots are usually poorly developed in the absence of K^+^ (Dolan & Davies, 2004; Tsay et al., 2011). However, in the present study, K^+^ did not seem to play an important role in the root development, as most of the measured root growth traits are not improved by the increased K^+^ or are even better without K^+^(Fig. 2). Gao (2010) has also reported that there was a negative linear correlation between the available potassium storage in the soil layer and the average height of *P. edulis*, yet there was no obvious correlation between the storage amount of available potassium and the diameter of *P. edulis*. When K^+^ concentration was elevated from 0 to 3 mmol/L, the N content of *P. edulis* reduced by 104.44% from treatments 1 to 7 when treated with 8 mmol/L NH_4_^+^ (Table 2). Similar findings have also been observed in barley and rice, where NH_4_^+^ absorption of barley roots decreases by 60% when external K^+^ increases from 0.1 mmol/L to 1.5 mmol/L (Szczerba et al., 2007; Balkos, Britto & Kronzucker, 2010). Therefore, we speculate that the inhibition of root growth due to the elevated exogenous K^+^ may be related to the reduced NH_4_^+^ uptake in moso bamboo.

In contrast to the competitive uptake between NH_4_^+^ and K^+^, the acquisition of K^+^ and NO_3_^−^ is usually cooperative (Tsay et al., 2011; Coskun, Britto & Kronzucker, 2017). The Dijkshoorn-Benzioni hypothesis has provided an intriguing example of the cooperative action of K ^+^ and NO_3_^−^ in plants, namely, that NO_3_^−^ is transported from roots to shoots in the xylem, using K^+^ as a counter ion (Benzioni, Vaadia & Lips, 1971; Coskun, Britto & Kronzucker, 2014; Dijkshoorn, 1958). It may be due to the charge balance of K^+^ and NO_3_^−^, which could be substituted by other cations, in particular Mg ^2+^ (Hagin, Olsen & Shaviv, 1990; Coskun, Britto & Kronzucker, 2014), low supply of K^+^ can activate NO_3_^−^ assimilation, reinforcing the NO_3_^−^ reduction in roots (Balkos, Britto & Kronzucker, 2010; Zhang et al., 2010). In this study, increasing K^+^ from 0.5 mmol/L to 1.5 mmol/L (treatments 4 and 6) with 8 mmol/L NO_3_^−^ treatment enhances the N content of moso bamboo seedlings by 53.85% (Table 2) in spite of a slightly growth inhibition of NH_4_^+^-preferring moso bamboo (Table 2), which might be related to the inhibited nitrate reductase activity due to the high endogenous NO_3_^−^ concentrations (Reddy & Menary, 1990). However, further studies are needed to determine the role of K in the response of moso bamboo to different N forms and the molecular physiological mechanisms of the low potassium requirements.

## Conclusions

N form and concentration play a significant role in the growth and N content of moso bamboo seedlings, while K^+^ concentration has a limited effect. The results from our experiments demonstrate improved growth of moso bamboo seedlings when treated with 8 mmol/L NH_4_^+^. Under elevated N concentrations, overall growth is inhibited though N content of the seedlings increases. In addition, root growth is inhibited with increasing K^+^ concentrations. Although moso bamboo seedlings display some level of NH_4_^+^-tolerance, high concentrations of NH_4_^+^can inhibit their growth, and increasing K^+^ concentration in the nutrient solution does not relieve the inhibitory effect of high NH_4_^+^ (32 mmol/L). Therefore, it is recommended that moso bamboo seedlings should be fed 8 mmol/L NH_4_^+^ fertilizer with a moderate K^+^ concentration.

##  Supplemental Information

10.7717/peerj.9938/supp-1Supplemental Information 1Phenotypes of *P. edulis* (moso bamboo) seedlings under different treatmentsThe seedlings showed greener, heathier leaves and less necrosis in the NH${}_{4}^{+}$ treatments 1, 3, 5 and 7 than the NO${}_{3}^{-}$ treatments 2, 4, 6 and 8, respectively. The image was taken after two months of different treatments. Bars = 30 cm.

10.7717/peerj.9938/supp-2 Supplemental Information 2 Original data of growth index under each treatment The raw data were used for range, variance and correlation analysis to compare the importance of N form, N and K concentrations on the growth of bamboo seedlings.

## Additional Information and Declarations

### Data Availability

The following information was supplied regarding data availability: The raw measurements are available in the Supplementary Files.
