# Supplementary figures and images for: Nitrogen form plays an important role in the growth of moso bamboo (Phyllostachys edulis) seedlings
Source: PeerJ. 2020 Sep 16;8:e9938. doi: 10.7717/peerj.9938 (PMC7501804; doi:10.7717/peerj.9938)

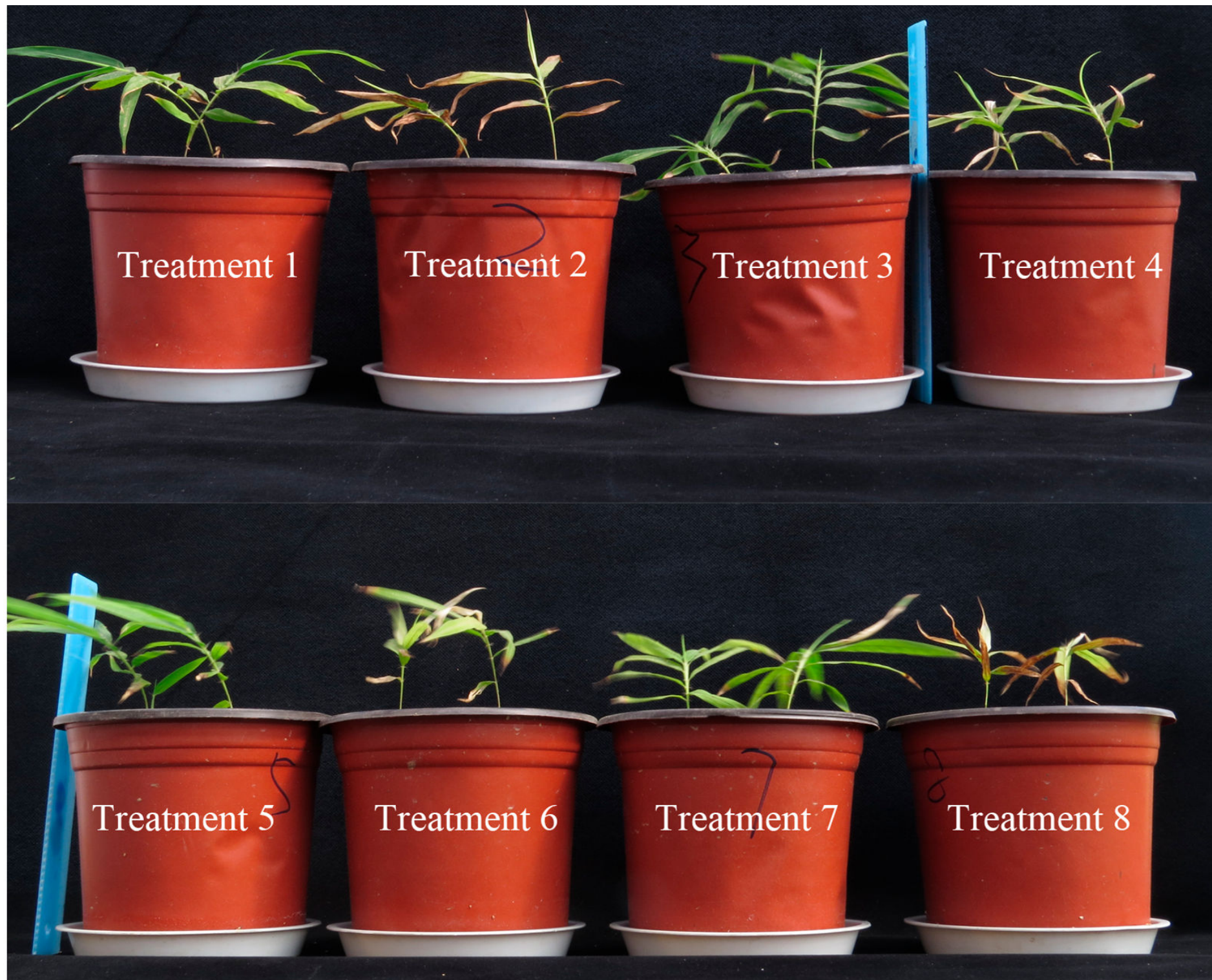

Supplement: Supplemental Information 1 — The seedlings showed greener, heathier leaves and less necrosis in the NH${}_{4}^{+}$4+ treatments 1, 3, 5 and 7 than the NO${}_{3}^{-}$3− treatments 2, 4, 6 and 8, respectively. The image was taken after two months of different treatments. Bars = 30 cm. [file peerj-08-9938-s001.pdf]
